# Effect of gender identity on the association between gender dysphoria and suicidality via appearance anxiety among transgender and gender-diverse young people: moderated mediation study

**DOI:** 10.1192/bjo.2024.18

**Published:** 2024-03-14

**Authors:** Jiaqi Li, Yi Feng, Yi Yu, Shicun Xu, Yuanyuan Wang

**Affiliations:** Key Laboratory of Brain, Cognition and Education Sciences, Ministry of Education, Guangzhou, China; and School of Psychology, Center for Studies of Psychological Application, and Guangdong Key Laboratory of Mental Health and Cognitive Science, South China Normal University, Guangzhou, China; Mental Health Center, Central University of Finance and Economics, Beijing, China; Northeast Asian Research Center, Jilin University, Changchun, China

**Keywords:** Gender dysphoria, appearance anxiety, suicidality, transgender, gender diversity

## Abstract

**Background:**

Gender dysphoria is associated with suicidality among transgender and gender-diverse (TGD) people. Gender dysphoria also results in a stress on appearance.

**Aims:**

The objectives of this study were to examine: (a) whether appearance anxiety mediates the effect of gender dysphoria on suicidality; and (b) whether gender identity moderates the mediating effect of appearance anxiety.

**Method:**

A total of 117 769 college and university students were recruited in this cross-sectional study from Jilin Province, China. After screening based on participants’ gender identity, 2352 TGD young people (aged from 15 to 25 years) were divided into three subgroups: female to male (FTM), male to female (MTF) and non-binary. Self-report inventories measured gender dysphoria, suicidality and appearance anxiety. A structural equation model was run to examine the relationships among TGD gender identity, gender dysphoria, appearance anxiety and suicidality.

**Results:**

Among TGD young people, gender dysphoria was significantly positively associated with suicidality (*β* = 0.15, 95% CI = 0.11–0.18, *P* < 0.001). Appearance anxiety partially mediated the association between gender dysphoria and suicidality (*β* = 0.07, 95% CI = 0.05–0.08, *P* < 0.001). Gender identity moderated the mediating effects: compared with individuals with FTM identity, among those with MTF and non-binary identities, gender dysphoria showed stronger positive effects on appearance anxiety, and appearance anxiety showed greater effects in mediating the association between gender dysphoria and suicidality.

**Conclusions:**

Among TGD young people, gender dysphoria is significantly associated with suicidality via appearance anxiety, with gender identity moderating the mediating effects. Diverse treatments should consider the heterogeneity of TGD subgroups, with the aim of limiting the tendency of gender dysphoria to trigger appearance anxiety, thus further buffering against the risk of suicide.

Suicide remains one of the major causes of death worldwide.^1^ The prevalence of suicidal ideation among the Chinese general young population ranges from 9.2 to 18%,^2,3^ and around 0.003 to 0.004% of this population complete suicide.^4^ The prevalence of overall suicidal ideation in Chinese transgender and gender-diverse (TGD) young people ranges from 43.03 to 56%, which is much higher than that in the general population.^5,6^ Thus, it is extremely important to investigate factors that influence suicidality, particularly in TGD young people.

Accumulating research reports the potential severity of gender dysphoria for these individuals, with prevalence ranging from 0.003 to 3.5%.^7,10^ Gender dysphoria is defined as a significant discomfort deriving from an incongruency between biological sex and perceived gender identity,^11^ and there is evidence that it increases the risk of suicide for TGD individuals.^12,13^ For example, an Amsterdam cohort study showed a higher suicide risk in TGD people with gender dysphoria than in the non-dysphoric cisgender population.^14^ In an *ex post facto* study based on a sample who consulted at a gender identity treatment unit, 48.3% of TGD individuals who complained of gender dysphoria reported suicidal ideation.^15^ In a study based in a Los Angeles paediatric setting among 101 TGD youth, 51% with gender dysphoria reported ever considering suicide, and 30% had attempted suicide at least once.^16^ Despite the established relationship between gender dysphoria and suicidality, it is crucial to investigate the mechanisms of this relationship among TGD young people.

Appearance anxiety refers to apprehension relating to one's physical appearance and others’ evaluations of it, comprising excessive appearance concern, appearance dissatisfaction and wishing to look good.^17^ In TGD young people, exorbitant body dissatisfaction is a common manifestation of appearance worries.^18,20^ For example, in a case report, a transgender woman's drive to lose weight was motivated by a desire to appear more ‘feminine’.^21^ Compared with cisgender counterparts, TGD individuals have higher levels of body image dissatisfaction, greater weight and shape concerns, and more eating problems.^20,22,23^ These manifestations all overlap with aspects of appearance anxiety. More importantly, TGD individuals with gender dysphoria are also prone to extreme dissatisfaction regarding their appearance.^12^ For example, US transgender individuals who reported higher levels of body incongruence had higher levels of body image dissatisfaction and anxiety.^24^ Owing to a continuing dissatisfaction with their primary and secondary sexual characteristics, individuals with gender dysphoria feel that they are in a vulnerable situation and have a persistent sense of mental disparities.^15^ Furthermore, published studies have indicated that appearance or body dissatisfaction can be positively correlated with suicidality among TGD samples.^12,22^ For instance, a birth-assigned female transitioning to male presenting with gender dysphoria stated, ‘I am so short. I am so ugly. I would be better off dead’.^12^ A prior study confirmed a significant positive correlation between suicidality and a desire for weight change among individuals with gender dysphoria.^22^ Even more significantly, transgender youth who endorsed a strong interest in weight loss or weight gain were more likely to have a history of suicide attempts (41%) than those without such interests (20%).^22^ Hence, it is reasonable to speculate that appearance anxiety may mediate the effects of gender dysphoria on suicidality among TGD young people.

There is diversity in the experience of appearance anxiety across gender identity subgroups among the TGD population.^18,25,26^ For instance, a cohort study found that transgender men had greater overall body dissatisfaction than transgender women. They reported more unhealthy body-related thoughts and behaviours, showing a higher possibility of medical therapy related to physical transition.^27^ Another Italian study indicated that accompanied by masculinity concerns, transgender men had higher scores on a drive for size, as well as more appearance anxiety than transgender women and non-binary people.^28^ A report of semi-structured interviews suggested that transgender men were more likely to suppress feminine bodily features by losing weight or by participating in more traditionally masculine activities to look more masculine,^29^ whereas a systematic literature review found that transgender women disliked more body parts than transgender men, being extremely dissatisfied with sex-specific body parts such as body hair and genitals.^18^ In a recent study, compared with their binary transgender counterparts, non-binary individuals were reported to have less body dissatisfaction with most body areas.^26^ Conversely, another study of young Chinese TGD adults reported lower body dissatisfaction among transgender men than transgender women and non-binary individuals.^25^ Earlier research in The Netherlands also found that natal men had more substantial wishes for physical transition than natal women (0.60% *v.* 0.25%), desiring to acquire an idealised feminine shape, particularly those who had gender dysphoria.^30^ Given these discrepancies, the mediating mechanism of appearance anxiety on the association between gender dysphoria and suicidality may differ between TGD subgroups. An in-depth understanding of the TGD gender identity and its influence on the mediating effect of appearance anxiety is crucial to buffer against suicidality among TGD young people. However, there is a lack of studies on the specific influences of TGD identity as a moderator.

Therefore, in the present study, we sought to explore the underlying mechanism of the association between gender dysphoria and suicidality in TGD young people, in particular, whether it was influenced by appearance anxiety and gender identity. Specifically, this study aimed to examine whether appearance anxiety mediates the effect of gender dysphoria on suicidality and whether gender identity moderates the mediating effect of appearance anxiety. Based on the above aims and literature review, we proposed two hypotheses. First, in TGD young people, gender dysphoria would be significantly positively related to suicidality, and appearance anxiety would mediate this relationship. Second, gender identity would moderate the mediating effect of appearance anxiety on the relationship between gender dysphoria and suicidality.

## Method

### Participants

This study was conducted on a subset of data extracted from a more extensive cross-sectional data-set, recruiting 117 769 students from 63 colleges and universities in Jilin Province, China, from October to November 2021. After receiving a QR code, the students completed an electronic questionnaire. The inclusion criteria for further analyses were: (a) aged from 15 years to 25 years; (b) being a student at a college or university in Jilin, China; and (3) understanding Chinese and providing informed consent.

Of the total 117 769 individuals who consented to participate in the survey, 28 427 were excluded owing to invalid data (*n* = 10), incorrect answers on the attention-check items (*n* = 21 541), inconsistent responses on gender identity questions (*n* = 2150), or invalid gender identity or sexual orientation responses (*n* = 4726). This left 89 342 participants whose data were entered and analysed, giving a completion rate of 75.86%.

The current study was granted ethical approval by the Ethics Committee of Jilin University, Changchun City, Jilin Province, China (no. 20210929; 11 October 2021), with ethical standards following the 1964 Declaration of Helsinki and its amendments in 2013. All participants were provided with information about the study, and electronic informed consent was obtained before the study commenced.

### Measurement

#### Sociodemographic information

Participants’ sociodemographic information was obtained using a questionnaire, including participants’ age, sex at birth, ethnicity, residence, whether they had siblings, and their relationships with their father and/or mother (on a five-point scale from ‘very bad’ to ‘very good’). Participants were also questioned about whether they had ever been diagnosed with generalised anxiety disorder, depressive disorder or substance misuse disorder. If they answered ‘Yes’, they were further asked whether they were currently taking any psychiatric medications.

#### Gender identity

Participants’ gender identity was measured by a one-item question: ‘If you could only choose one option, which of the following would you say best describes you?’ Answers comprised: (a) man; (b) woman; (c) transgender: female to male (birth sex female, affirmed identity as male; FTM); (d) transgender: male to female (birth sex male, affirmed identity as female; MTF); (e) non-binary gender/genderqueer (identity does not exclusively fall into binary male or female categories). In this study, all participants with gender identities other than the cisgender man and woman identities (where gender identity aligns with birth sex) composed the data subset of TGD young people. This measurement method was consistent with those used in other studies that also combined multiple non-cisgender identities into one general group.^5,31^

#### Gender dysphoria

Gender dysphoria was measured by the Utrecht Gender Dysphoria Scale-Gender Spectrum.^32^ It consists of 18 self-report items measuring general gender dysphoria in two dimensions, and each item is scored on a five-point scale ranging from 0 (‘disagree completely’) to 5 (‘agree completely’). All item scores were summed to give a total score, with a higher total score indicating more severe gender dysphoria. The Chinese version of this scale has been validated in the Chinese population (Cronbach's *α* = 0.90).^33^

#### Appearance anxiety

Appearance anxiety was measured by the Appearance Anxiety Scale-Brief Version,^17^ which was designed to measure the degree to which individuals worry about their appearance. It contains 14 items rated on a five-point Likert scale from 1 (‘never’) to 5 (‘always’). The items on the scale were divided into positive subscale items (e.g. ‘I am very satisfied with my appearance’) and negative subscale items (e.g. ‘I was nervous about my appearance’). The positive subscale items were reverse-scored and together with the scores of the negative subscale were summed to a total score. A higher total score indicated a higher degree of appearance anxiety. The scale was translated into Chinese, showing an acceptable internal consistency coefficient of 0.82.^34^ In this study, the scale showed a good Cronbach's *α* of 0.87.

#### Suicidality

Participants’ suicidality was measured by the Suicidal Behaviors Questionnaire-Revised.^35^ A total of four items were used to assess whether participants self-reported suicidality (e.g. ‘Have you ever thought about or attempted to kill yourself?’). Scores from all items were summed together to form a total score for this scale, with a higher total score indicating more severe suicidality. The Chinese version of this scale has been validated among diverse samples.^36,37^

### Statistical analysis

Analyses were conducted on 2352 TGD samples after screening based on participants’ gender identities, divided into three subgroups (FTM, MTF and non-binary). First, descriptive analysis was applied to the sociodemographic information of the TGD young people. One-way analysis of variance (ANOVA) and *post hoc* comparisons were used to compare the differences among the three subgroups concerning continuous variables, and chi-squared tests were used for categorical variables. Second, after controlling the covariates (i.e. sex assigned at birth, relationships with father or mother, diagnosis of generalised anxiety disorder or depressive disorder, and taking psychiatric medication), we set gender dysphoria as the independent variable, suicidality as the dependent variable and appearance anxiety as the mediating variable and then analysed the mediating effects of appearance anxiety on the association between gender dysphoria and suicidality. Then, we set gender identity as the moderating variable. Given that gender identity was categorical, we used the dummy coding method with FTM and non-binary as reference groups and conducted two moderated mediation analyses of the same structure equation model. In this way, we explored the differences in mediating roles among the FTM, MTF and non-binary groups. Model fitness was evaluated using the χ^2^-degree of freedom ratio (χ^2^ / d.f.), comparative fit index (CFI), Tucker–Lewis index (TLI), root mean square error of approximation (RMSEA) and standardised root mean residual (SRMR). The acceptable criteria for the model were set to CFI > 0.90,^38^ TLI > 0.90,^39^ RMSEA < 0.08^40^ and SRMR < 0.08.^40^ All statistical analyses were carried out using SPSS 25.0, Mplus 8.3 and R software 4.3.1. The significance level was set to *P* < 0.05 for a two-tailed test.

## Results

### Differences among MTF, FTM and non-binary groups

According to the one-way ANOVA results, significant differences in gender dysphoria (*F* = 5.40, *P* = 0.005) and suicidality (*F* = 50.30, *P* < 0.001) existed among the FTM, MTF and non-binary groups. Further *post hoc* comparison (using the Games–Howell test) indicated that the severity of gender dysphoria was lowest in the MTF group. Regarding suicidality, the MTF group had the lowest scores, whereas the non-binary group had the highest (Table 1).

**Table 1:** Sociodemographic characteristics of transgender individuals^a^

Variables	Total (*N* = 2352)	FTM (*N* = 1223)	MTF (*N* = 538)	Non-binary (*N* = 591)	*F*/χ^2^	*η^2^*/*V*	*Post hoc* comparison
	*M* (s.d.)				*F*	*η^2^*	
Age, years	19.58 (1.87)	19.59 (1.87)	19.50 (1.99)	19.64 (1.75)	0.73	0.001	
Relationship with father	4.05 (0.95)	4.11 (0.95)	4.11 (0.96)	3.87 (0.96)	**14**.25***	0.012	1 > 3; 2 > 3
Relationship with mother	4.41 (0.78)	4.43 (0.78)	4.51 (0.73)	4.28 (0.82)	**12**.78***	0.011	1 > 3; 2 > 3
Gender dysphoria	49.52 (12.12)	49.93 (11.82)	48.01 (13.55)	50.03 (11.24)	**5**.40**	0.005	1 > 2; 3 > 2
Appearance anxiety	43.14 (10.48)	43.09 (9.80)	43.19 (10.59)	43.19 (11.70)	0.027	0.001	
Suicidality	6.61 (3.79)	6.44 (3.72)	5.66 (3.46)	7.82 (3.91)	50.30***	0.041	3 > 1; 3 > 2; 1 > 2
	*n* (%)				*χ^2^*	*V*	
Sex					**1853**.33***	0.888	
Male	668 (28.4%)	0 (0%)	538 (100%)	130 (22.0%)			2 > 1; 2 > 3; 3 > 1
Female	1684 (71.6%)	1223 (100%)	0 (0%)	461 (78.0%)			1 > 2; 1 > 3; 3 > 2
Only-child status					**18**.80***	0.089	
Only child	1129 (48.0%)	536 (43.8%)	291 (54.1%)	302 (51.1%)			2 > 1; 3 > 1
Having siblings	1223 (52.0%)	687 (56.2%)	247 (45.9%)	289 (48.9%)			1 > 2; 1 > 3
Ethnicity					4.23	0.042	
Han	2066 (87.8%)	1058 (86.5%)	480 (89.2%)	528 (89.3%)			
Others	286 (12.2%)	165 (13.5%)	58 (10.8%)	63 (10.7%)			
Residence					**16**.29***	0.083	
Urban	1334 (56.7%)	669 (54.7%)	288 (53.5%)	377 (63.8%)			3 > 1; 3 > 2
Rural	1018 (43.3%)	554 (45.3%)	250 (46.5%)	214 (36.2%)			1 > 3; 2 > 3
Depressive disorder (yes)	253 (10.8%)	112 (9.2%)	50 (9.3%)	91 (15.4%)	**17**.72***	0.087	3 > 1; 3 > 2
Generalised anxiety disorder (yes)	82 (3.5%)	31 (2.5%)	17 (3.2%)	34 (5.8%)	**12**.49**	0.073	3 > 1; 2 > 1
Substance use disorder (yes)	7 (0.3%)	1 (0.1%)	4 (0.7%)	2 (0.3%)	5.56	0.049	
Taking psychiatric medication (yes)	64 (2.7%)	28 (2.3%)	13 (2.4%)	23 (3.9%)	4.11	0.042	

FTM, female to male; MTF, male to female. The bold text refers to significant differences existing in groups on specific variables.

a. Results of one-way ANOVA tests and χ^2^ tests for between-group differences and related effect size estimates (partial *η*^2^ and Cramer's *V* tests) are presented.

**P < 0.01, ***P < 0.001.

### The mediating role of appearance anxiety

**Fig. 1:**
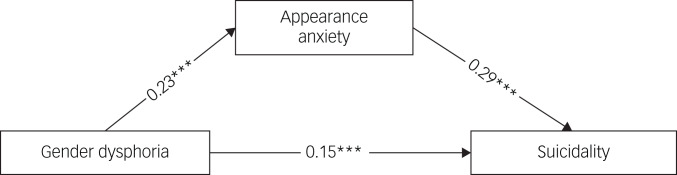
Mediation effect of appearance anxiety on the association between gender dysphoria and suicidality. Model fitting: root mean square error of approximation, 0.044, comparative fit index, 0.989, Tucker–Lewis index, 0.944, standardised root mean residual, 0.021. ***P < 0.001.

Gender dysphoria was significantly positively associated with suicidality (*β* = 0.15, 95% CI = 0.11–0.18, *P* < 0.001) and positively related to appearance anxiety (*β* = 0.23, 95% CI = 0.19–0.27, *P* < 0.001). In addition, appearance anxiety was positively linked to suicidality (*β* = 0.29, 95% CI = 0.25–0.33, *P* < 0.001) (Fig. 1). The results also suggested that appearance anxiety partially mediated the relationship between gender dysphoria and suicidality (*β* = 0.07, 95% CI = 0.05–0.08, *P* < 0.001), accounting for 31.3% of the overall effect.

### The moderating effect of gender identity on appearance anxiety

**Fig. 2:**
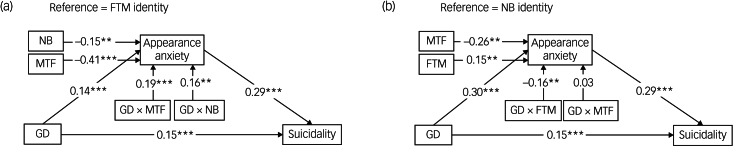
The moderated mediation model with gender identity as the moderator. Gender identity was assessed in ternary form as ‘FTM’, ‘MTF’ and ‘non-binary’. GD, gender dysphoria; FTM, female to male; MTF, male to female; NB, non-binary. **P < 0.01; ***P < 0.001.

The differences in moderation effects among the TGD subgroups were statistically significant in this study. When FTM identity was set as the reference, gender dysphoria was significantly positively associated with appearance anxiety in the MTF (*b* = 0.19, 95% CI = 0.11–0.27, *P* < 0.001) and non-binary (*b* = 0.16, 95% CI = 0.05–0.27, *P* = 0.006) identity groups (Fig. 2(a)). When non-binary was set as the reference, gender dysphoria was significantly negatively associated with appearance anxiety in the FTM group (*b* = −0.16, 95% CI = −0.27–0.05, *P* = 0.006), but there was no significant association in the MTF group (*b* = 0.03, 95% CI = −0.09–0.15, *P* = 0.602) (Fig. 2(b)).

These findings indicated that the moderating effect of FTM identity on the path from gender dysphoria to appearance anxiety was significantly different from that of MTF (Wald test: *χ^2^* = 15.95, *P* < 0.001) or non-binary identity (Wald test: *χ^2^* = 6.74, *P* = 0.009), with no significant difference between the latter two subgroups.

### The moderating effect of gender identity on mediation

We next explored the indirect effects with gender identity as a moderator and found that gender dysphoria retained the same positive linkage with suicidality. The effect of gender dysphoria on suicidality through appearance anxiety was significant in the three gender identity subgroups, and the mediating effect of appearance anxiety on the association between gender dysphoria and suicidality was significant in all three subgroups. In the FTM group, the mediating effect was 0.04 (95% CI = 0.02–0.06, *P* < 0.001); in the MTF group, it was 0.10 (95% CI = 0.07–0.12, *P* < 0.001); and in the non-binary group, it was 0.09 (95% CI = 0.06–0.12, *P* < 0.001) (Table 2).

**Table 2:** Path coefficients and indirect effect in different gender groups

Path^a^	Mediation model		Moderated mediation model with GD as a moderator					
			FTM		MTF		Non-binary	
	β	95% CI	β	95% CI	β	95% CI	β	95% CI
Regression paths								
GD → suicidality	0.15***	[0.11, 0.18]	0.15***	[0.11, 0.20]	0.11**	[0.03, 0.18]	0.17***	[0.10, 0.24]
GD → appearance anxiety	0.23***	[0.19, 0.27]	0.15***	[0.10, 0.21]	0.36***	[0.29, 0.43]	0.24***	[0.16, 0.31]
Appearance anxiety → suicidality	0.29***	[0.25, 0.33]	0.29***	[0.25, 0.34]	0.33***	[0.26, 0.41]	0.28***	[0.21, 0.35]
Indirect effects	0.07***	[0.05, 0.08]	0.04***	[0.02, 0.06]	0.10***	[0.07, 0.12]	0.09***	[0.06, 0.12]

GD, gender dysphoria; FTM, female to male; MTF, male to female; β, standardised coefficient.

a. The results of regression paths were obtained by multigroup analysis, and the indirect effects were calculated using a moderated mediation model.

**P < 0.01, ***P < 0.001.

**Fig. 3:**
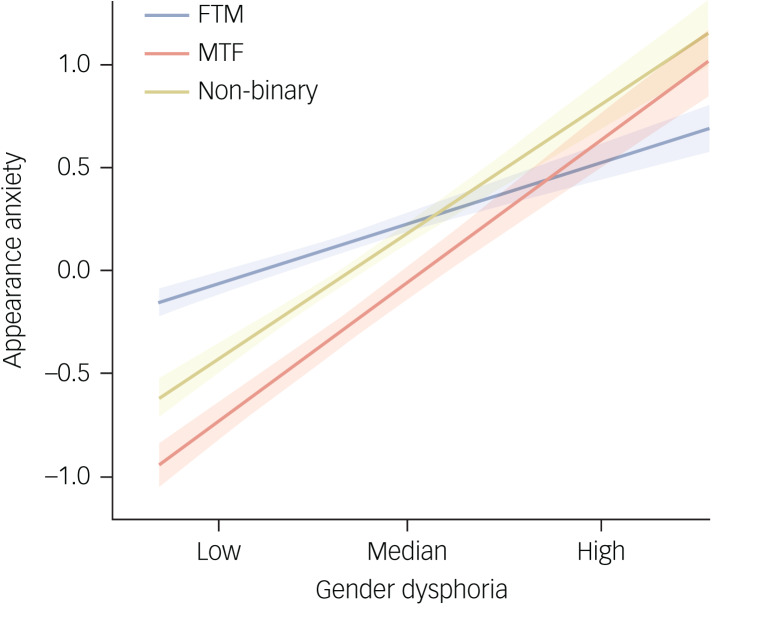
Gender identity as a moderator between gender dysphoria and appearance anxiety. FTM, female to male; MTF, male to female. Both gender dysphoria and appearance anxiety were standardised.

Simple slope analysis further indicated that in the MTF and non-binary groups, the severity of appearance anxiety would increase with increasing gender dysphoria. There was also a significant increase in the FTM group, although the rate of increase was the smoothest among three subgroups (Fig. 3).

Taken together, the results shown in Figs. 2 and 3 and Table 2 show that in terms of the path from gender dysphoria to appearance anxiety and the overall mediating effect, FTM identity exerted significantly lower moderating effects than MTF and non-binary identities, with no significant differences between the latter two identities.

## Discussion

This study investigated, for the first time, the mediating effect of appearance anxiety on the association between gender dysphoria and suicidality among TGD young people and further explored the moderating role of gender identity. The most novel finding was that MTF and non-binary identity were more likely to positively influence the path from gender dysphoria to appearance anxiety, exhibiting higher indirect effects on suicidality, whereas the moderating effect was significantly lower among FTM individuals.

Among TGD young people in this study, gender dysphoria was significantly positively associated with suicidality, and this effect was partially mediated by appearance anxiety. This finding validated the first hypothesis in of present study and was consistent with previous studies reporting that individuals with gender dysphoria exhibit excessive concern about appearance, appearance dissatisfaction, and a wish for good looks,^19,25^ composing three aspects of appearance anxiety. These aspects of appearance anxiety influence the suicide risk of TGD populations to a certain degree.

First, TGD individuals frequently experience gender dysphoria as a unique proximal stressor that cisgender individuals generally do not encounter.^41,42^ For example, 68.3% of TGD individuals in a recent study had experienced severe gender dysphoria, and they also focused on external appearance standards of conventional binary gender expression, such as masculinity or femininity.^43^ Such excessive body concerns probably put them at suicide-related risk.^22^ Second, a queer theory framework suggests that stereotypical gender expectations in a cultural context can exacerbate young TGD adults’ worries about their appearance and may contribute to body dissatisfaction.^19^ Aligning with societal standards of beauty (e.g. for women to be thin and men to be muscular) has been regarded as a source of body dissatisfaction.^44^ Finally, TGD young people frequently desire an attractive appearance and ideal body, hoping to please themselves or obtain a positive evaluation from others.^45,46^ This may generate appearance-based anxiety.^47,48^ Thus, these three aspects surrounding appearance probably add to the severity of appearance anxiety in TGD young people, with subsequent effects on suicidality.

Notably, we found that gender identity had a significant moderating role in the mediation model, confirming the second hypothesis. The present study focused on TGD subgroups, enabling us to show that MTF identity more crucially affected the connection between gender dysphoria and suicidality through appearance anxiety, compared with FTM identity. There are several potential explanations for this finding. As stated in previous research, societal tolerance may be higher towards masculine women than feminine men, and natal males may be less likely to be accepted in terms of female gender expression without physical transition.^30^ Thus, MTF individuals who have gender dysphoria may encounter stronger feelings of appearance dissatisfaction owing to fear of being perceived as a man that is not masculine enough. Moreover, regarding the ideal female body, several MTF individuals may internalise the same dominant cultural standards that some cisgender females abide by.^49^ Given that women are under greater pressure to adhere to an ideal physical appearance derived from the same structure of social norms,^50^ MTF young people unavoidably experience higher physical and appearance stress under the influence of desired female appearance. In addition, MTF individuals aiming for appearance congruency report high pressure to engage in intense self-surveillance of their bodies in order not to be read as transgender,^51^ resulting in higher rates of hormone use or silicone injections than FTM individuals.^49,52^ This type of body surveillance has also been found to be significantly associated with appearance anxiety.^53^ Therefore, when MTF young people experience gender dysphoria, they may have additional appearance anxiety and be at higher risk of suicidality.

Gender dysphoria had a more significant positive effect on appearance anxiety in non-binary individuals compared with FTM individuals, and a higher indirect effect on suicidality. Previous results have indicated that non-binary individuals understand gender or sex more broadly than transgender male youth^54^ and attain resilience and joy from their non-binary identity.^55^ However, in this study, appearance anxiety was aggravated more by increasing gender dysphoria in non-binary young people compared with those with FTM identity. This may be attributed to non-binary individuals’ desire to express their gender androgynously.^43^ Some non-binary individuals with female birth-assigned sex may retain feminine characteristics (e.g. breasts) and may also engage in modification of visible markers of gender such as bodybuilding or packing the groin region to obtain a stereotypically male contour.^56^ Nevertheless, such androgynous expression may add additional pressure to counter the societal gender-binary norms of appearance for non-binary young people.^43^ Moreover, as they may not align with binary gender expression, non-binary individuals sometimes experience prejudice by others, claiming their gender identities are fake, invented or exist online only.^57^ Some non-binary individuals have also reported that they face the stigma of being read as not ‘trans enough’ to justify a transgender identity label.^58^ The adverse mental influence from such prejudice and stigma from ‘both sides’ may be aggravated by gender dysphoria,^59^ resulting in dissatisfaction with appearance. More importantly, when seeking gender dysphoria treatment, non-binary individuals probably experience external pressure in the binary-medical context.^60,61^ A recent study reported that because a substantial proportion of the transgender and medical communities regard gender dysphoria as binaristic and embodied, non-binary individuals experience pressure to access non-normative binary embodiment desires.^61^ These experiences of pressure probably frustrated them and added to their worries about their appearance, resulting in a stronger indirect effect on suicidality for non-binary individuals.

The findings of this study enrich empirical evidence of the minority stress theory, which posits that gender dysphoria as a proximal stressor can interact with other stressors, eventually influencing individuals’ mental well-being.^42^ Further, we found that TGD young people's gender dysphoria was positively linked to suicidality via the interaction with appearance anxiety. In addition, findings indicated that in comparison with gender dysphoric FTM young people, those with MTF and non-binary identities were more likely to progress from appearance anxiety to suicidality. Hence, identifying the co-occurrence of appearance anxiety in gender dysphoric TGD young people can provide a perspective for buffering against their suicide risk. It is likely that in gender-dysphoric young people identifying as MTF and non-binary, targeted intervention in this regard could be more likely to reduce the risk of suicide.

There are several limitations to note in this study. First, the study was cross-sectional and cannot be used to draw causal conclusions. Longitudinal or experimental studies are required for causal interpretation of the present findings. Second, as most studies on appearance or body expression among TGD individuals have focused on body dissatisfaction, there was less consideration of the other two aspects of appearance anxiety (i.e. excessive appearance concerns and wishing for good looks) in the comparisons of TGD subgroups. Thus, whether diverse types of appearance anxiety have different influences also needs to be considered in the future. Third, we did not directly measure whether the TGD individuals in this study abided by the binary societal norm of gender expression and its consequences; this should be explored in future work. Fourth, this study did not find any change in the direct relationship between appearance anxiety and suicidality when setting gender identity as a moderator. Whether this result was a consequence of the limited sample should be investigated. Fifth, the majority of TGD individuals in this study were natal females, of Han ethnicity, and college students from urban areas in the north-east of China. This limits the generalisability of the results to the wider population. Thus, studies recruiting participants from a broader range of regions and with more diverse demographic characteristics should be conducted. In addition, the self-reported data may have included errors due to social desirability or recall bias. Therefore, multiple methods should be used to replicate the results, such as interviews, teacher reports and peer nominations (i.e. a method to assess group dynamics and behaviours exhibited in peer-rating situations). Finally, as gender dysphoria is related to other psychiatric disorders (e.g. body dysmorphic disorder and personality disorders),^59,62,63^ it will be necessary to explore possible comorbidity factors relating to suicidality in future studies.

In conclusion, this study demonstrated the mediating role of appearance anxiety in the effect that gender dysphoria exerts on suicidality among TGD young people. In addition, TGD gender identity could moderate this mediating effect. In particular, gender dysphoria had a stronger positive effect on appearance anxiety, and appearance anxiety had a greater mediating role in the effect of gender dysphoria on suicidality in the MTF and non-binary groups compared with the FTM group. These novel findings indicate that various treatments for alleviating gender dysphoria in TGD subgroups should be considered, including addressing the tendency for gender dysphoria to trigger appearance anxiety, to further buffer against suicidality.

## Data Availability

The data-set for this study is available to potential collaborators upon reasonable request.
